# The complete mitochondrial genome of pronghorn spiny lobster *Panulirus penicillatus* (Olivier, 1791)

**DOI:** 10.1080/23802359.2020.1852899

**Published:** 2021-01-17

**Authors:** Hongtao Liu, Guangyuan Xia

**Affiliations:** a Key Laboratory of Utilization and Conservation for Tropical Marine Bioresources, Ministry of Education, Hainan Tropical Ocean University, Sanya, China; b Hainan Provincial Key Laboratory of Tropical Maricultural Technologies, Hainan Academy of Ocean and Fisheries Sciences, Haikou, China

**Keywords:** *Panulirus penicillatus*, mitochondrial genome, phylogenetic analysis

## Abstract

In this paper, we determined and characterized the complete mitochondrial genome of Pronghorn spiny lobster *Panulirus penicillatus* for the first time from South China Sea. The *P. penicillatus* mitogenome is 15,671 bp long, and consists of 22 tRNA genes, 2 rRNA genes, 13 protein-coding genes (PCGs), and 1 control region. The nucleotide composition of *P. penicillatus* mitogenome is significantly biased (A, G, T, and C was 33.62, 13.32, 32.31, and 20.75%, respectively) with A + T contents of 65.93%. Almost PCGs used a standard initiation codon or stop codon, except COX2, ND3, ND4 and ND1 were terminated with an incomplete stop codon T and ND5 ended with TA. One microsatellite (C)_12_ was identified in the control region of *P. penicillatus* mitogenome sequences. Phylogenetic tree showed that *P. penicillatus* was first clustered with *P. polyphagus* and *P. versicolor*.

*Panulirus penicillatus*, commonly known as the pronghorn spiny lobster or double-spined rock lobster, is an important commercial species belonging to the family Palinuridae. It is probably the widest global distribution of any species of spiny lobster which known to occur in tropical and sub-tropical areas of the Indo-Pacific region from East Africa and the Red Sea across to Pacific Mexico and Central America (Holthuis 1991; Vaitheeswaran 2018). *Panulirus penicillatus* is not gregarious and is nocturnal, usually found in shallow watersat a depth range of 1–4 m in surf-zones of coral reefs and large rocky outcroppings. It is heavily exploited for food throughout its wide range, this does not seem to have had a large impact on total populations. Wide-ranging stock assessments and biological reference points such as population ecology, growth, and minimum suitable catch size, sexual maturity and reproduction season have been examined to support a modest fishery of *P. penicillatus* (Ebert and Ford 1986; Plaut 1993; Hogarth and Barratt 1996; Chauvet and Coutures 2001; Szuwalski et al. 2016). Early studies mainly focused on the developmental and reproductive biology of *P. penicillatus*. Recently, the researchers have paid more attention to its genetics including the genetic isolation between the western and eastern Pacific populations, genetic diversity and population structure, range-wide phytogeography and development of compound microsatellite markers (Chow et al. 2011; Abdullah et al. 2014, 2017; Iacchei et al. 2016). Morphological differences among phyllosoma larvae from the different Pacific populations also showed that there are significant geographic differences same as the above genetic analysis (Matsuda et al. 2019a, 2019b).

The specimens of *P. penicillatus* were collected from Qionghai, China (N19°18′48.37″, E110°40′20.21″), and stored in the marine crustacean specimen room (C20191119PP) in Qionghai research base of Hainan Academy of Ocean and Fisheries Sciences. The libraries with an average length of 350 bp were constructed using the NexteraXT DNA Library Preparation Kit, and sequencing was performed on the Illumina Novaseq platform (Total Genomics Solution Limited, SZHT) with the 150 bp average length of the generated reads. The complete mitochondrial genome of *P. longipes* were assembled with 5.91 G clean reads using the de novo assembler SPAdes 3.11.0, and annotated using the MITOS (http://mitos.bioinf.uni-leipzig.de/index.py). Based on nucleotide sequences of 20 Achelata species mitogenome available in the GenBank (Table S2), a phylogenetic analysis was carried out using IQ-TREE(Nguyen et al. 2015) v1.6.12 to investigate the evolution position of *P. penicillatus* using the maximum–likelihood (ML) method with 1000 bootstrap replicates.

The whole mitogenome of *P. penicillatus* (Table S1) is 15,671 bp in size (GenBank Accession No. MT533488). The base content was 32.80% A, 12.01% G, 32.63% T, and 22.56% C. The 65.43% of (A + T) showed great preference to AT. It consists of 22 tRNA genes, 2 rRNA genes, 13 protein-coding genes (PCGs), and 1 control region. Four PCGs (*ND1, ND4, ND4L*, and *ND5*), eight tRNA genes and two rRNA genes were located on the light strand, the others were encoded by the heavy strand.

The 22 tRNA genes in *P. penicillatus* mitogenome vary in length from 63 to 72 bp. tRNA-Leu and tRNA-Ser both have two type copies respectively. The 12S rRNA is 863 bp and located between tRNA-Val and the control region, and the 16S rRNA is 1291 bp, located between tRNA-Val and tRNA-Leu. All 13 PCGs use a normal initiation codon ATN or TTG. Simultaneously, eight PCGs were terminated with a usual stop codon in addition five PCGs using a normal stop codon (COX2, ND3, ND4 and ND1 use a single T; ND5 use TA). The control region is 731 bp, located between 12S rRNA and tRNA-Ile. Interestingly, we identified one microsatellite (SSR) in *P. penicillatus* mitogenome using MISA (Beier et al. 2017), a (C)_12_ is located in the control region which was also found similar in some closely related species with different number of repetitions (Figure S1).

**Figure 1.**
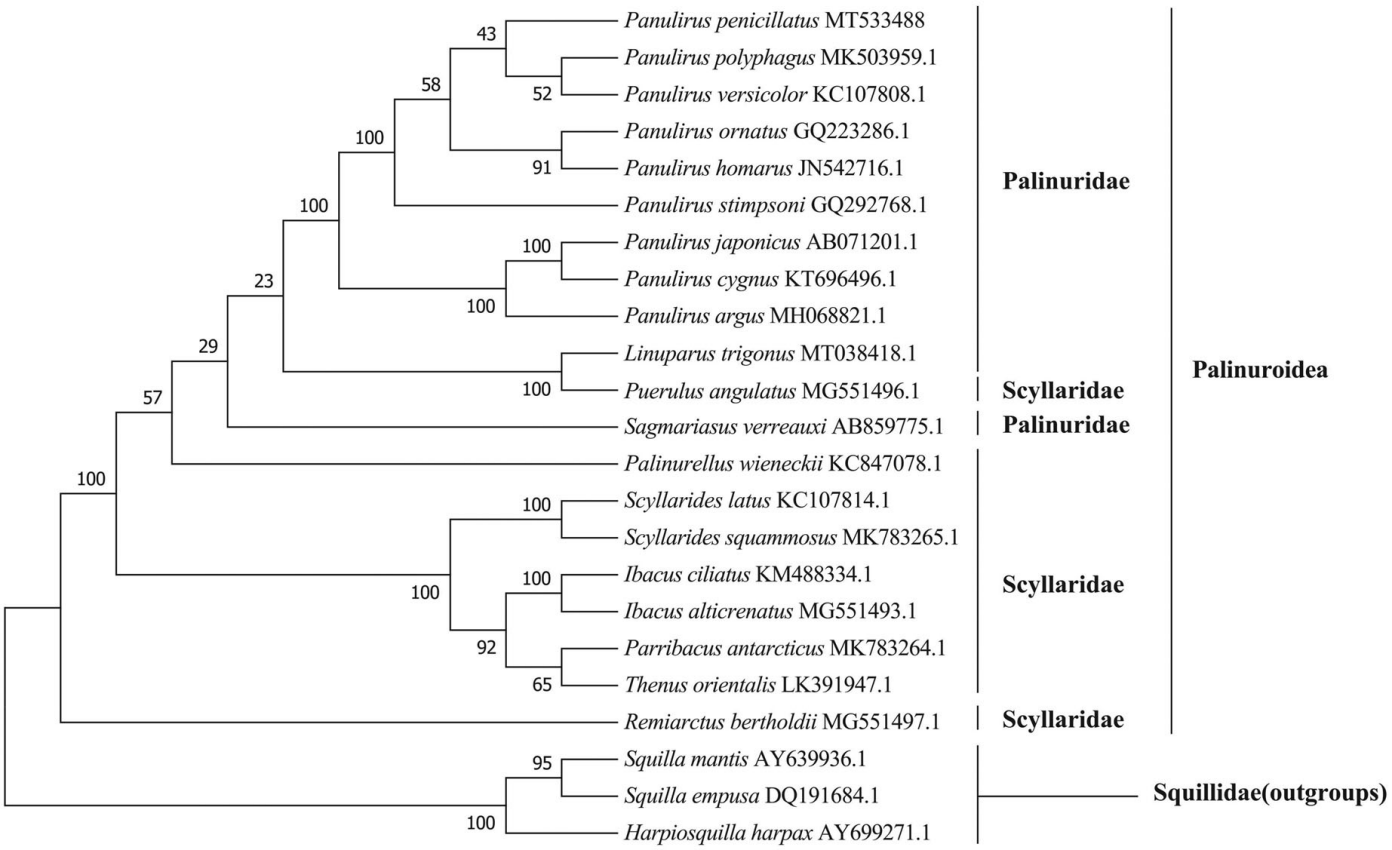
The maximum likelihood tree of *P. penicillatus* and 22 other species based on 13 PCGs.

The phylogenetic tree (Figure 1) showed that *P. penicillatus* was formed a clade by first clustering with *P. polyphagus* and *P. versicolor*, and further clarified the phylogenetic relationships of the species of lobsters in the family Palinuridae.

*Harpiosquilla harpax, Squilla empusa*, and *Squilla mantis* were used as outgroups.

## Supplementary Material

Supplemental Material

## Data Availability

The data that support the findings of this study are available in “figshare” at https://doi.org/10.6084/m9.figshare.12812081.v1.
