# Supplementary material for: The complete mitochondrial genome of pronghorn spiny lobster Panulirus penicillatus (Olivier, 1791)
Source: Mitochondrial DNA B Resour. 2021 Jan 17;6(1):148–50. doi: 10.1080/23802359.2020.1852899 (PMC7832592; doi:10.1080/23802359.2020.1852899)
Supplement: Supplemental Material [file TMDN_A_1852899_SM0231.zip › Supplementary_Table_S2._List_of_organisms_used_in_the_phylogenetic_analyses_of_Panulirus_penicillatus.docx]

Supplementary Table S2. List of organisms used in the phylogenetic analyses.

| Organism | Family | Length | Accession number |
| --- | --- | --- | --- |
| *Panulirus penicillatus* | Palinuridae | 15,671 | MT533488.1 |
| *Scyllarides latus* | Scyllaridae | 15,663 | KC107814.1 |
| *Scyllarides squammosus* | Scyllaridae | 15.644 | MK783265.1 |
| *Parribacus antarcticus* | Scyllaridae | 15,806 | MK783264.1 |
| *Ibacus alticrenatus* | Scyllaridae | 15,762 | MG551493.1 |
| *Ibacus ciliatus* | Scyllaridae | 15,696 | KM488334.1 |
| *Thenus orientalis* | Scyllaridae | 16,826 | LK391947.1 |
| *Remiarctus bertholdii* | Scyllaridae | 15,807 | MG551497.1 |
| *Palinurellus wieneckii* | Synaxidae | 15,699 | KC847078.1 |
| *Panulirus ornatus* | Palinuridae | 16,105 | GQ223286.1 |
| *Panulirus versicolor* | Palinuridae | 15,767 | KC107808.1 |
| *Panulirus argus* | Palinuridae | 15,739 | MH068821.1 |
| *Panulirus cygnus* | Palinuridae | 15,724 | KT696496.1 |
| *Panulirus japonicus* | Palinuridae | 15,717 | AB071201.1 |
| *Panulirus stimpsoni* | Palinuridae | 15,677 | GQ292768.1 |
| *Panulirus homarus* | Palinuridae | 15,665 | JN542716.1 |
| *Panulirus polyphagus* | Palinuridae | 15,707 | MK503959.1 |
| *Puerulus angulatus* | Palinuridae | 15,688 | MG551496.1 |
| *Sagmariasus verreauxi* | Palinuridae | 15,470 | AB859775.1 |
| *Linuparus trigonus* | Palinuridae | 15,949 | MT038418.1 |
| *Squilla mantis* | Squillidae | 15,994 | AY639936.1 |
| *Squilla empusa* | Squillidae | 15,828 | DQ191684.1 |
| *Harpiosquilla harpax* | Squillidae | 15,714 | AY699271.1 |
