# Supplementary material for: The complete mitochondrial genome of pronghorn spiny lobster Panulirus penicillatus (Olivier, 1791)
Source: Mitochondrial DNA B Resour. 2021 Jan 17;6(1):148–50. doi: 10.1080/23802359.2020.1852899 (PMC7832592; doi:10.1080/23802359.2020.1852899)
Supplement: Supplemental Material [file TMDN_A_1852899_SM0231.zip › Supplydata_Table_S1._List_of_the_Panulirus_penicillatus_mitogenome_annotation.docx]

**Supplydata table 1. List of the *Panulirus penicillatus* mitogenome annotation**

| **Start** | **End** | **Length** | **Strand** | **Start codon** | **Stop codon** | **Gene** |
| --- | --- | --- | --- | --- | --- | --- |
| 1 | 66 | 66 | + |  |  | tRNA-Ile |
| 64 | 132 | 69 | - |  |  | tRNA-Gln |
| 142 | 208 | 67 | + |  |  | tRNA-Met |
| 230 | 1210 | 981 | + | ATT | TAA | nad2 |
| 1209 | 1277 | 69 | + |  |  | tRNA-Trp |
| 1277 | 1342 | 66 | - |  |  | tRNA-Cys |
| 1346 | 1410 | 65 | - |  |  | tRNA-Tyr |
| 1408 | 2949 | 1542 | + | ATC | TAA | cox1 |
| 2945 | 3008 | 64 | + |  |  | tRNA-Leu |
| 3014 | 3701 | 688 | + | ATG | T | cox2 |
| 3702 | 3766 | 65 | + |  |  | tRNA-Lys |
| 3780 | 3842 | 63 | + |  |  | tRNA-Asp |
| 3843 | 3998 | 156 | + | ATG | TGA | atp8 |
| 3995 | 4672 | 678 | + | ATG | TAA | atp6 |
| 4672 | 5463 | 792 | + | ATG | TAA | cox3 |
| 5463 | 5528 | 66 | + |  |  | tRNA-Gly |
| 5538 | 5880 | 343 | + | ATC | T | nad3 |
| 5881 | 5943 | 63 | + |  |  | tRNA-Ala |
| 5946 | 6009 | 64 | + |  |  | tRNA-Arg |
| 6013 | 6078 | 66 | + |  |  | tRNA-Asn |
| 6079 | 6146 | 68 | + |  |  | tRNA-Ser |
| 6146 | 6216 | 71 | + |  |  | tRNA-Glu |
| 6225 | 6290 | 66 | - |  |  | tRNA-Phe |
| 6293 | 7968 | 1676 | - | TTG | TA | nad5 |
| 8020 | 8084 | 65 | - |  |  | tRNA-His |
| 8100 | 9423 | 1324 | - | ATG | T | nad4 |
| 9417 | 9677 | 261 | - | ATG | TAA | nad4l |
| 9722 | 9788 | 67 | + |  |  | tRNA-Thr |
| 9789 | 9855 | 67 | - |  |  | tRNA-Pro |
| 9864 | 10373 | 510 | + | ATC | TAA | nad6 |
| 10386 | 11510 | 1125 | + | ATT | TGA | cob |
| 11509 | 11576 | 68 | + |  |  | tRNA-Ser |
| 11643 | 12543 | 901 | - | ATT | T | nad1 |
| 12589 | 12658 | 70 | - |  |  | tRNA-Leu |
| 12669 | 13959 | 1291 | - |  |  | 16S |
| 14006 | 14077 | 72 | - |  |  | tRNA-Val |
| 14078 | 14940 | 863 | - |  |  | 12S |
