# Supplementary figures and images for: The complete mitochondrial genome of pronghorn spiny lobster Panulirus penicillatus (Olivier, 1791)
Source: Mitochondrial DNA B Resour. 2021 Jan 17;6(1):148–50. doi: 10.1080/23802359.2020.1852899 (PMC7832592; doi:10.1080/23802359.2020.1852899)

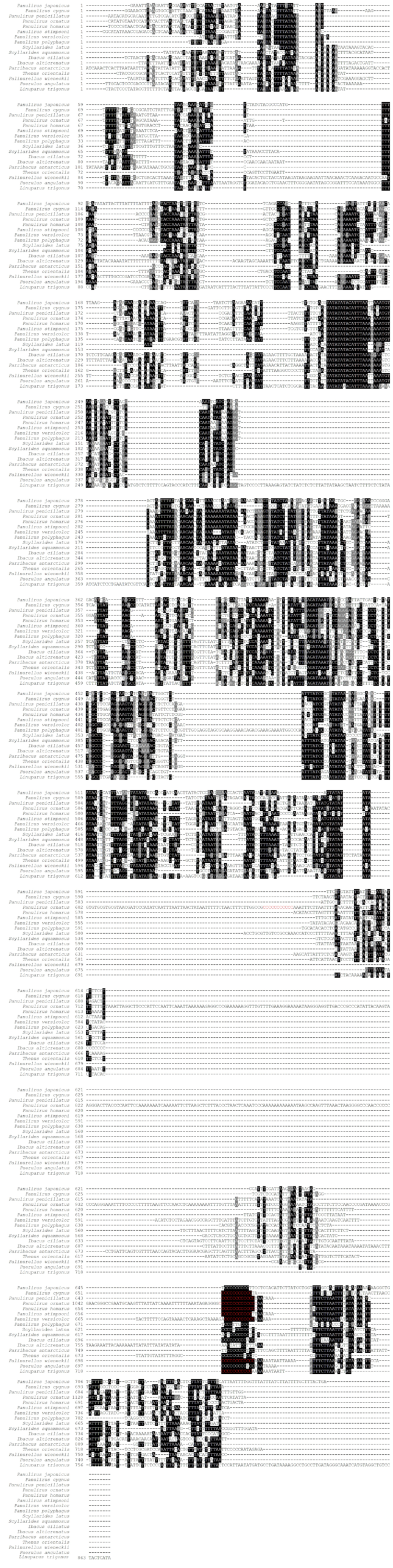

Supplement: Supplemental Material [file TMDN_A_1852899_SM0231.zip › Supplementary_Figure_S1._The_graphical_alignment_representation_analyses_of_the_Panulirus_penicillatus_D_loop_region.jpg]
